# Synergistic drug combinations and machine learning for drug repurposing in chordoma

**DOI:** 10.1038/s41598-020-70026-w

**Published:** 2020-07-31

**Authors:** Edward Anderson, Tammy M. Havener, Kimberley M. Zorn, Daniel H. Foil, Thomas R. Lane, Stephen J. Capuzzi, Dave Morris, Anthony J. Hickey, David H. Drewry, Sean Ekins

**Affiliations:** 1 UNC Catalyst for Rare Diseases, Eshelman School of Pharmacy, University of North Carolina at Chapel Hill, Chapel Hill, NC USA; 2 Collaborations Pharmaceuticals, Inc., 840 Main Campus Drive, Lab 3510, Raleigh, NC USA; 3 RTI International, Research Triangle Park, NC USA; 4 Structural Genomics Consortium, UNC Eshelman School of Pharmacy, University of North Carolina at Chapel Hill, Chapel Hill, NC 27599 USA

**Keywords:** Computational biology and bioinformatics, Drug discovery, Diseases, Oncology

## Abstract

Chordoma is a devastating rare cancer that affects one in a million people. With a mean-survival of just 6 years and no approved medicines, the primary treatments are surgery and radiation. In order to speed new medicines to chordoma patients, a drug repurposing strategy represents an attractive approach. Drugs that have already advanced through human clinical safety trials have the potential to be approved more quickly than de novo discovered medicines on new targets. We have taken two strategies to enable this: (1) generated and validated machine learning models of chordoma inhibition and screened compounds of interest in vitro. (2) Tested combinations of approved kinase inhibitors already being individually evaluated for chordoma. Several published studies of compounds screened against chordoma cell lines were used to generate Bayesian Machine learning models which were then used to score compounds selected from the NIH NCATS industry-provided assets. Out of these compounds, the mTOR inhibitor AZD2014, was the most potent against chordoma cell lines (IC_50_ 0.35 µM U-CH1 and 0.61 µM U-CH2). Several studies have shown the importance of the mTOR signaling pathway in chordoma and suggest it as a promising avenue for targeted therapy. Additionally, two currently FDA approved drugs, afatinib and palbociclib (EGFR and CDK4/6 inhibitors, respectively) demonstrated synergy in vitro (CI_50_ = 0.43) while AZD2014 and afatanib also showed synergy (CI_50_ = 0.41) against a chordoma cell in vitro. These findings may be of interest clinically, and this in vitro*-* and in silico approach could also be applied to other rare cancers.

## Introduction

Chordoma is a rare cancer that occurs in the bones of the skull base and spine which is part of a larger class of tumors known as sarcomas. Chordoma tumors develop from cells of the notochord, an embryonic structure that facilitates development of the spine^1^. The notochord disappears when the fetus is about 8 weeks old, but some notochord cells remain in the bones of the spine and skull base^2^. This is a rare occurrence, but when they do, these cells can turn into chordoma. A chordoma tumor usually grows slowly without symptoms for years before diagnosis, which is often in the 5^th^ and 6^th^ decades of life (although it can occur at any age). Studies have demonstrated that skull base chordomas are observed more often in children, whilst spinal chordomas are more frequently observed later in life^2,3^. It has also been described that when chordomas metastasize they frequently distribute to the lungs, liver, bones, or lymph nodes. This occurs in 30 to 40 percent of people where the tumor metastasizes to other parts of the body^2^. At this point in time there are no known environmental, dietary or lifestyle risk factors for this rare type of cancer. Chordomas often occur at random with no direct inherited genetic trait, however familial cases can be caused by duplications of the brachyury gene^4^. A SNP in the brachyury gene occurs in 95 percent of people with this tumor^5,6^, and furthermore, chordomas have been reported at a higher incidence in children diagnosed with the genetic disease Tuberous Sclerosis Complex (TSC)^7^. With a mean-survival rate of just 6 years and poor response to current medications, surgical resection is the main course of treatment^2^. Patients therefore need new and effective drugs to expand their treatment options and improve survival rates.

Chordoma tumors, which occur in both pediatric and adult populations, are known to overexpress multiple kinases^4^. Kinases are a family of ~ 500 proteins, collectively known as the kinome, integral for a multitude of cellular functions relevant to cancer pathogenesis. In a 2013 study^8^, a tissue microarray containing 58 chordomas was used to examine the expression of the kinases PDGFR-α, PDGFR-β, EGFR, c-Met, c-Kit, pAKT, mTOR, and HER2. Most tumors were positive by immunohistochemistry for PDGFR-α (92%), PDGFR-β (85%), c-Kit (77%), c-Met (96%), pAKT (82%), mTOR (56%), HER2 (24%), and EGFR (26%), yet imatinib, an FDA-approved drug that inhibits PDGFR-α, PDGFR-β, and c-Kit, has shown little to no efficacy in chordoma in vivo models^9^. A body of such molecular, preclinical, and clinical evidence of interest to chordoma oncogenesis has begun to emerge for several kinases: Epidermal Growth Factor Receptor (EGFR), Cyclin-dependent kinase 4 (CDK4), Cyclin-dependent kinase 6 (CDK6) and the mammalian target of rapamycin (mTOR). These kinases are well-studied in the field of oncology, with several FDA-approved drugs on the market targeting each kinase and they may serve as drug repurposing candidates for the treatment of chordoma.

Drug repurposing or repositioning is an approach whereby new therapeutic uses for existing drugs or clinical candidates are identified^10-14^. High throughput screens, virtual screening or serendipitous observations are employed to enable drug repurposing^13^. For example we have previously identified approved drugs active against the Ebola virus^15^ and Chagas Disease^16^ using Bayesian and other machine learning models. In addition, there are several ongoing efforts to demonstrate new uses for molecules that have been through clinical trials for other uses but were subsequently shelved. One such example is the NIH NCATS industry-provided assets that could be potentially repurposed (https://ncats.nih.gov/ntu/assets/current). We have now developed a strategy for virtual screening such compounds then testing in vitro and will describe this approach applied to chordoma.

**Figure 1:**
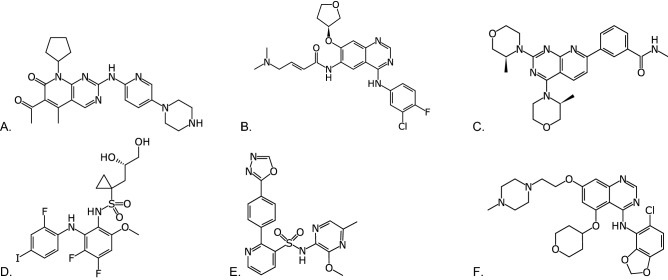
Molecule structures evaluated in this study. (**A**) Palbociclib (CDK4/6) and (**B**) Afatinib (EGFR), (**C**) AZD2014 (targets mTOR1 and mTOR2), (**D**) RDEA119 (targets MEK1/2), (**E**) AZD4054 (targets endothelin A receptor). (**F**) AZD0530 (Src inhibitor). Images created with Mobile Molecular DataSheet (Molecular Materials Informatics, Inc., Montreal Canada).

Further, two FDA-approved kinase inhibitor drugs—palbociclib, a breast cancer drug, and afatinib (Fig. 1A,B), a non-small cell lung carcinoma drug—have shown equally robust efficacy in patient derived xenograft (PDX) and cell-line derived xenograft (CDX) models of chordoma. Palbociclib^17^ and afatinib^18^ were designed to specifically target different kinases, CDK4/6 and EGFR, respectively. The utility of each in preclinical chordoma models implies that multiple oncogenic biological pathways may drive chordoma as is true in many other cancers.

We hypothesized that combinations of clinical kinase inhibitors in chordoma models and patients may act additively or synergistically by dampening oncogenic signaling in multiple pathways. Both primary targets and secondary targets can play a role in this. A combination therapy of afatinib and palbociclib is of particular interest but there may also be many other potential combinations of kinase inhibitors. Chordoma PDX and CDX mouse models respond equally well to afatinib and palbociclib, though these drugs target divergent and minimally overlapping regions of the kinome^19^. A combination of EGFR/CDK inhibitors, *i.e.*, afatinib/palbociclib, may target multiple oncogenic signaling pathways simultaneously. Following the same rationale, we now evaluate the in vitro efficacy of EGFR/CDK inhibitor combinations prior to future in vivo PDX and CDX mouse model studies. We envision that these proposed studies will enable and support future drug combination chordoma clinical trials.

## Results

### Machine learning models for chordoma drug discovery

**Figure 2:**
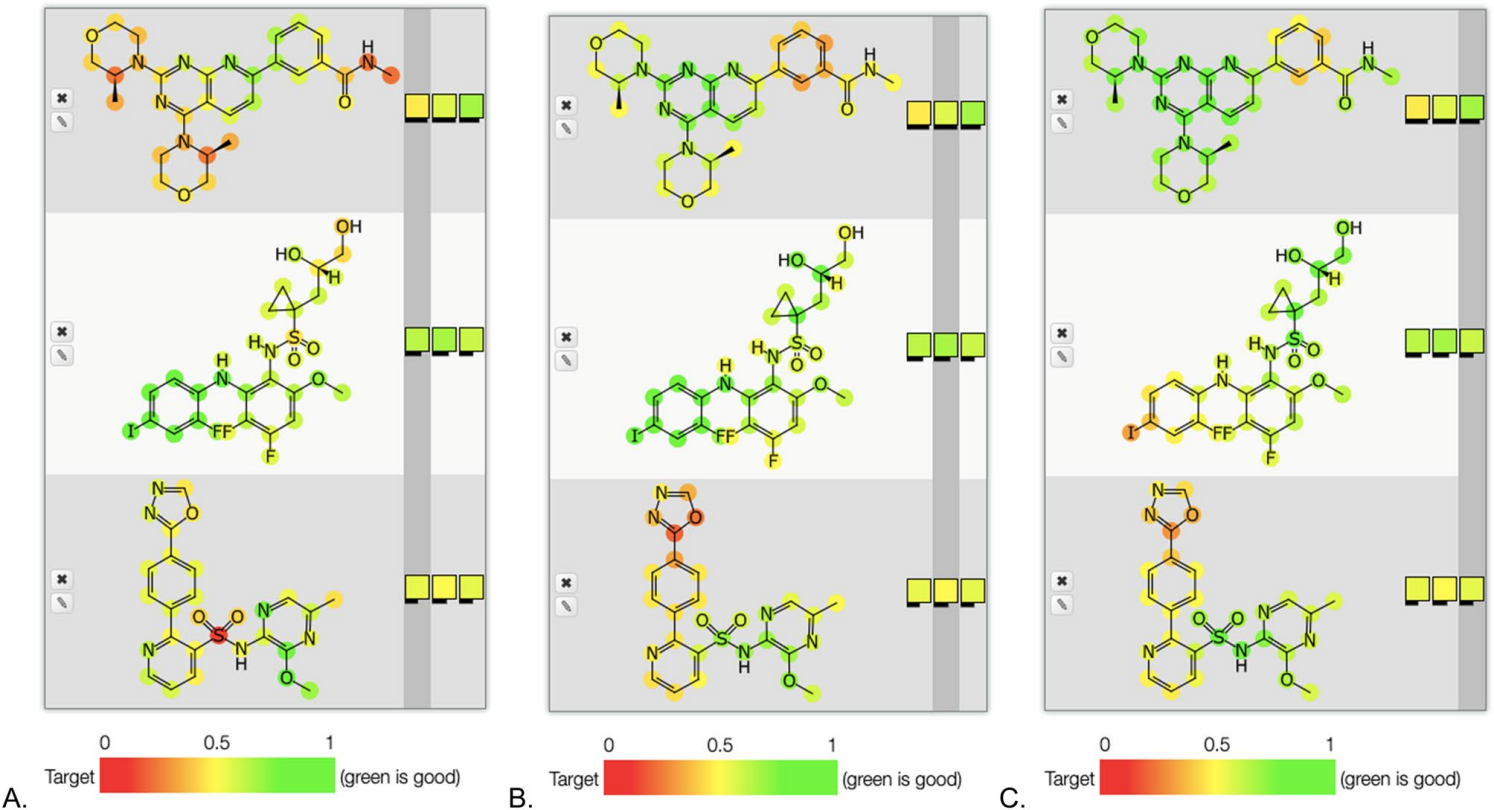
Examples of atom highlighting derived from Bayesian models. The molecules not in each model were tested with (**A**) Broad, (**B**) Broad + EGFR and (**C**) EGFR models. Molecules shown are: AZD2014 (top), RDEA119 (middle), AZD4054 (bottom) on each panel. Images were generated in Assay Central (Collaborations Pharmaceuticals, Inc.).

Several recently published studies of compounds screened against chordoma cell lines^20,21^ were used to generate Bayesian machine learning models with our Assay Central software^10,12,22-29^. In one published chordoma study 1097 compounds were screened against 3 chordoma cell lines (U-CH1, U-CH2, MUG-Chor1) and 27 had chordoma selective cytotoxicity^20^ and many of these were EGFR inhibitors. A more recent study from the Broad Institute and collaborators profiled 459 compounds in 4 chordoma cell lines (JHC7, MUG-Chor1, U-CH1, UM-Chor1) and identified 28 potent antiproliferative compounds with several kinase inhibitors including CDK 7/12/13 inhibitors^21^. These data sets (which for ease we have called EGFR or Broad) were used either separately or combined to generate machine learning models that were evaluated with fivefold cross validation (Table 1, Figure S1). These models were also used to score the 3 molecules not in these training sets (Table 2 and Fig. 1C,D) and their scores would suggest the value of testing them in vitro. The atom coloring feature helps to suggest which molecule features correspond to activity with a particular model [e.g. with the EGFR model AZD2014 has a large area of the molecule colored green (favorable for activity) while the other molecules have fewer green colored atoms (Fig. 2A–C)].

**Table 1:** Chordoma Bayesian model statistics.

Model name	Actives	Size	ROC	F1	Kappa	MCC	Domain
Broad	150	454	0.67	0.54	0.23	0.25	0.36
Broad + EGFR	289	1486	0.81	0.52	0.36	0.39	0.33
EGFR	147	1064	0.84	0.44	0.30	0.37	0.28

Datasets were named as “Broad”^21^ and “EGFR”^20^, and underwent curation to remove problematic molecules before model building. Data represent fivefold cross validation. The “Broad” dataset is named as such because the data came from a chordoma screen at the Broad Institute. The “EGFR” data set is so named because it came from a paper that highlighted the activity of EGFR compounds in chordoma and is not meant to construe a dataset made up entirely of EGFR compounds. Both datasets contain a wide variety of compounds that inhibit a broad range of targets.

**Table 2:** Bayesian Machine Learning predictions for chordoma activity for compounds not in the training sets.

Compound	Broad Bayesian score	Broad model applicability	Broad/EGFR Bayesian score	Broad/EGFR model applicability	EGFR Bayesian score	EGFR model applicability
AZD2014	0.46	0.91	0.56	0.96	0.67	0.82
RDEA11	0.62	0.66	0.66	0.67	0.60	0.57
AZD4054	0.53	0.53	0.50	0.60	0.55	0.55

Datasets were named as Broad^21^ and EGFR^20^ and models are described in Table 1. Model applicability assesses the portion of fragments overlapping with the training set molecules, higher values indicate more fragments overlapping with the training set. The “score” is the prediction score, a measure of probability of activity with higher values being desirable.

### In vitro studies

**Figure 3:**
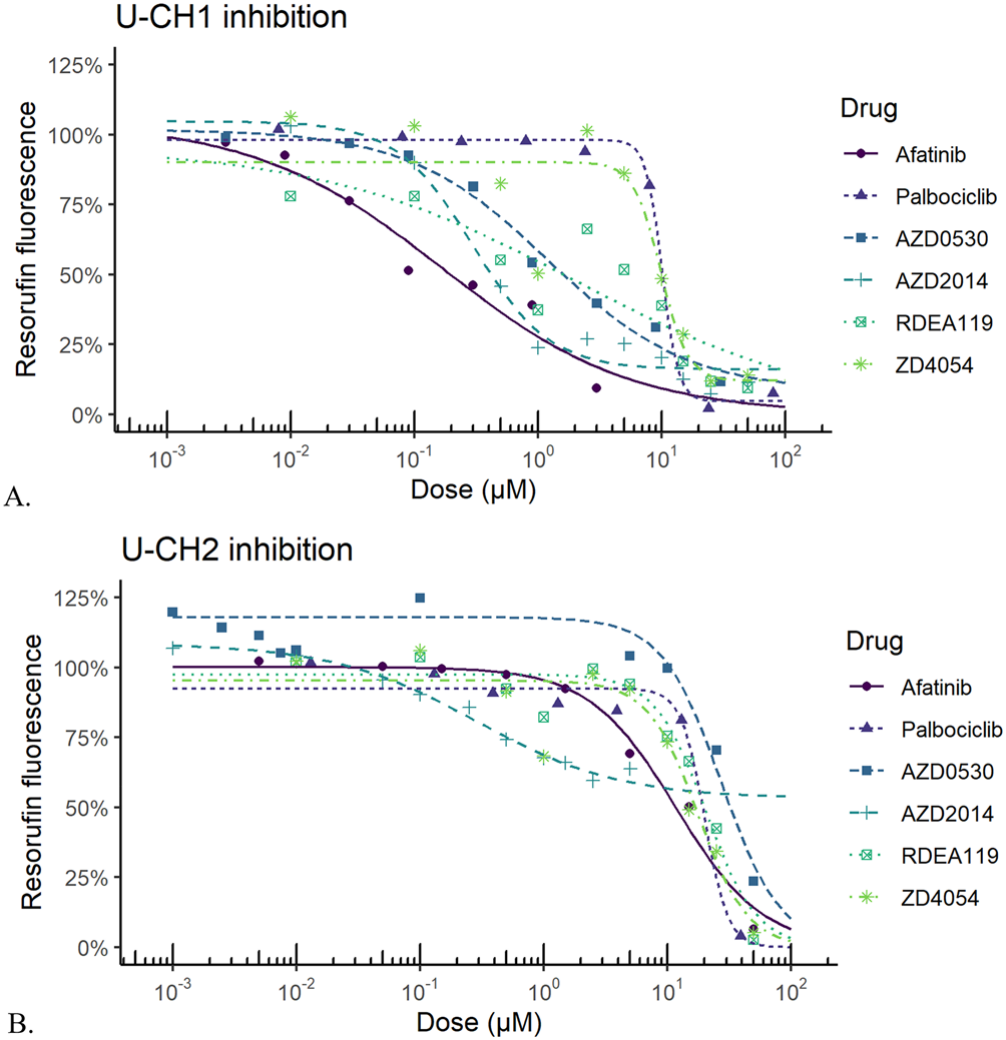
Representative dose response curves for compounds tested against chordoma cell lines in vitro. Molecules were tested in (**A**) U-CH1 cells and (**B**) U-CH2 cells. Graphs produced with R^66^ version 3.6.2 (https://cran.r-project.org/), with the following packages: drc 3.0–6 for general dose–response curve fitting and analysis, medrc 1.1–0 for mixed-effects modeling of dose–response data, multcomp 1.4–11 for multiple comparison testing, and ggplot2 3.2.1 for plotting.

We tested AZD2014 (Fig. 1C), RDEA119 (Fig. 1D), AZD4054 (Fig. 1E) AZD0530, (Fig. 1F) in UCH1 and UCH2 cell lines, along with known chordoma inhibitors afatinib and palbociclib, using reduction of resazurin to resarufin as an assay for metabolic activity of chordoma cell lines. As previously reported^18^, afatinib is a potent inhibitor of U-CH1, but not U-CH2. The Bayesian model we have termed ‘EGFR’ correctly predicted the rank order of these 3 compounds not in the training sets (Table 2). Of the drugs predicted by our model, AZD2014 was the most potent against both cell lines (IC_50_ 0.35 µM U-CH1 and 0.61 µM U-CH2, Table 3 and Fig. 3A,B), though maximal inhibition of U-CH2 cells was limited. AZD0530 and RDEA119 also showed moderate potency against U-CH1 (IC_50_ 1.5 µM and 2.4 µM, respectively) but not U-CH2. AZD4054 had low potency, but even at 11 µM this was unexpected given that its target, the endothelin A receptor, has no known functions in chordoma.

**Table 3:** Summary of resazurin reduction assays for chordoma cell lines.

Drug	U-CH1				U-CH2			
	IC_50_ (µM, absolute)	IC_50_ (µM, relative)	Maximal inhibition (%)	n	IC_50_ (µM, absolute)	IC_50_ (µM, relative)	Maximal inhibition (%)	n
Afatinib	0.54	0.38	93	6	9.4	8.8	98	7
Palbociclib	11.4	10.8	96	6	20.8	19.5	94	6
RDEA119	–	2.4	59	2	24.5	24.4	94	4
AZD0530	2.7	1.5	85	5	29.4	26.6	100	3
AZD2014	0.94	0.35	72	7	–	0.61	53	8
AZD4054	11.9	11.0	84	2	18.8	19.2	100	2

Data generated includes geometric mean IC_50_, and arithmetic mean of maximal inhibition. Absolute IC_50_ is the dose that reduces resazurin fluorescence by 50%, relative to positive and negative controls. Relative IC_50_ is the dose that has 50% of the maximal inhibition for a given drug. Where values are absent this represents the drug never reaching 50% inhibition in several replicate experiments.

### In vitro studies—combination studies of approved drugs

**Figure 4:**
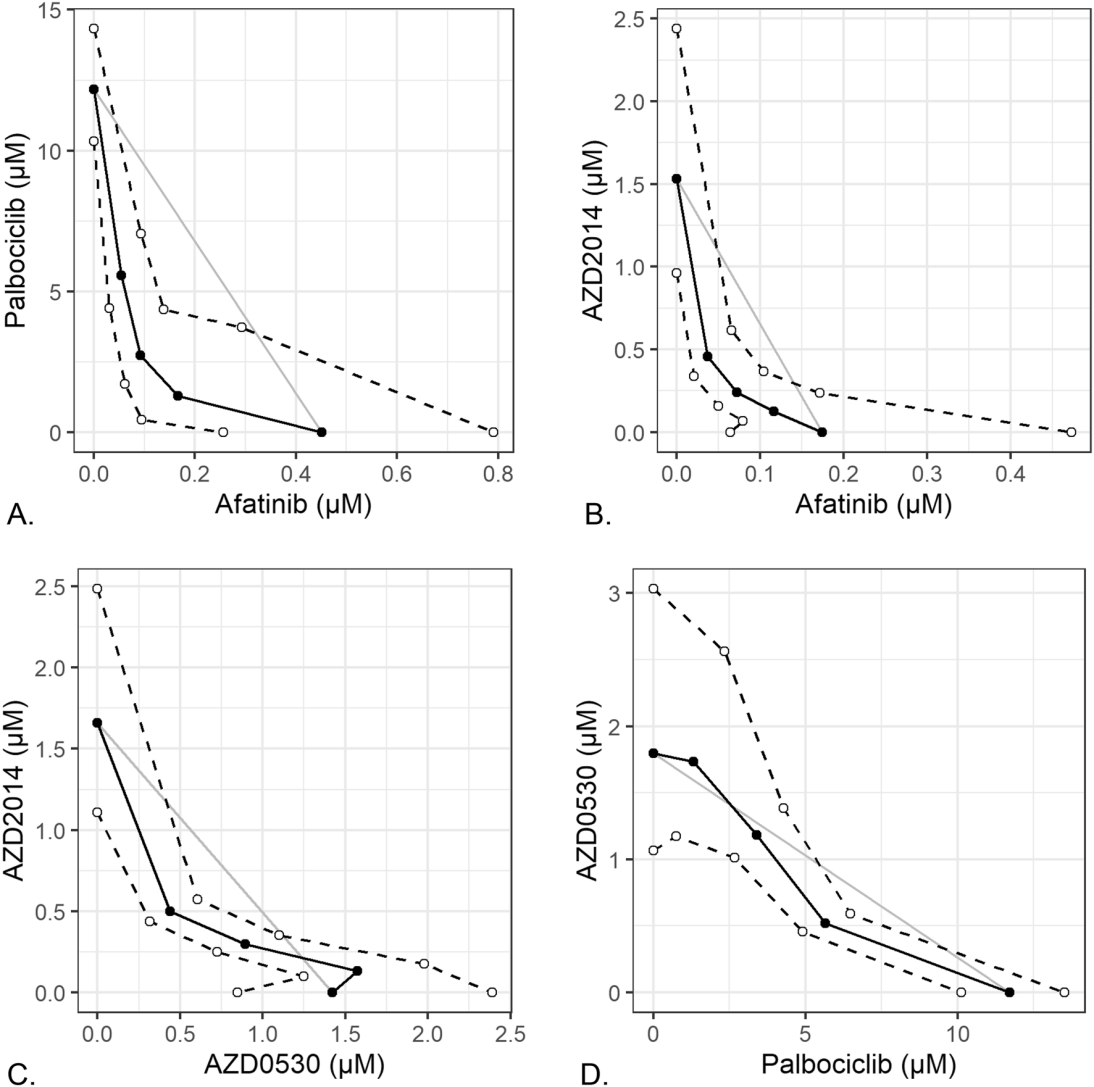
Isobolograms for combinations of compounds demonstrating significant synergy. (**A**) palbociclib and afatanib, (**B**) AZD2014 and afatanib, (**C**) AZD2014 and AZD0530, (**D**) AZD0530 and palbociclib. The Loewe Additivity isobole is shown as the diagonal grey line, representing the expected IC_50_ of non-interacting combinations of drugs. Measured absolute IC_50_ of drug combinations (black line and solid dots) represent the isobole of dose combinations with equivalent activity, with dashed lines and open dots indicating the 95% confidence band. Points below and to the left of the isobole indicate a synergistic drug interaction. Graphs produced with R^66^ version 3.6.2 (https://cran.r-project.org/), with the following packages: drc 3.0–6 for general dose–response curve fitting and analysis, medrc 1.1–0 for mixed-effects modeling of dose–response data, multcomp 1.4–11 for multiple comparison testing, and ggplot2 3.2.1 for plotting.

Next we tested the combinations of drugs used in this study to determine whether inhibition of multiple biological pathways would result in synergistic effects on chordoma cell lines. In order to determine this we calculated the combination index (CI) for each of the pairs of drugs mixed at different ratios, where CI < 1 indicates a synergistic effect. For each of the pairs of drugs used, CI was then calculated using five eight-point dose curves, each with a constant ratio of the two drugs. In U-CH1 cells, we observed substantial synergy between afatinib and palbociclib (CI = 0.43, 95% CL 0.28–0.66, Fig. 4A, Table 4). We also observed substantial synergy between afatinib and AZD2014 (CI = 0.41, 95% CL 0.34–0.78, Fig. 4B). The combination of AZD0530 and AZD2014 showed more modest synergy (CI = 0.77, 95% CI 0.67–0.88, Fig. 4C), while the combination of palbociclib and AZD0530 had weak but detectable synergy (CI50 = 0.61, 95% CI 0.49–0.77, Fig. 4D). However, in U-CH2 cells, we did not observe synergistic effects in any tested combinations (Table 5).

**Table 4:** Summary of drug combinations in U-CH1 cells.

Effect level	Drug 1	Drug 2	Ratio	CI	UCL	LCL
50%	Afatinib	Palbociclib	**0.0094:1**	**0.58**	**0.79**	**0.43**
			**0.038:1**	**0.43**	**0.66**	**0.28**
			**0.15:1**	**0.48**	**0.96**	**0.25**
50%	Afatinib	AZD0530	0.025:1	0.98	2.92	0.34
			0.1:1	1.10	2.63	0.48
			0.4:1	1.05	2.36	0.53
50%	Afatinib	AZD2014	**0.075:1**	**0.51**	**0.78**	**0.34**
			**0.3:1**	**0.57**	**0.84**	**0.39**
			1.2:1	0.75	1.14	0.50
50%	Palbociclib	AZD0530	0.67:1	1.08	1.62	0.72
			2.7:1	0.95	1.14	0.79
			**11:1**	**0.77**	**0.88**	**0.67**
75%	Palbociclib	AZD2014	2:1	1.432	2.289	0.895
			8:1	1.398	1.911	1.024
			32:1	1.248	1.478	1.053
50%	AZD0530	AZD2014	**0.75:1**	**0.61**	**0.77**	**0.49**
			**3:1**	**0.81**	**0.99**	**0.66**
			12:1	1.19	1.50	0.94

Each CI was calculated at the specified effect level, along with upper and lower confidence limits (UCL and LCL). Combinations with significant synergy are indicated in bold.

**Table 5:** Summary of drug combinations in U-CH2 cells.

Effect level	Drug 1	Drug 2	Ratio	CI	UCL	LCL
50%	Afatinib	Palbociclib	0.096:1	0.68	1.45	0.33
			0.38:1	0.52	1.76	0.25
			1.5:1	0.59	5.47	0.40
50%	Afatinib	RDEA119	0.074:1	1.04	2.25	0.49
			0.29:1	0.98	2.78	0.48
			1.2:1	0.53	> 10	0.36
75%	Afatinib	AZD2014	0.62:1	0.71	2.37	0.22
			2.5:1	0.83	1.41	0.49
			10:1	0.75	1.14	0.50
75%	Palbociclib	RDEA119	0.19:1	1.00	> 10	0.33
			0.76:1	0.57	5.60	0.06
			3.1:1	0.38	> 10	0.03
75%	RDEA119	AZD2014	2.1:1	1.77	> 10	0.08
			8.5:1	3.02	> 10	1.16
			34:1	1.63	4.97	0.54

Each CI was calculated at the specified effect level, along with upper and lower confidence limits (UCL and LCL). No combinations had significant synergy.

## Discussion

Epidermal Growth Factor Receptor (EGFR) is a receptor tyrosine kinase (RTK)^30^. Activation of EGFR leads to the phosphorylation of proteins in downstream signaling pathways, including the PI3k-Akt-mTOR and RAS-RAF-MEK-MAPK pathways^30^. Both of these pathways are critical in regulating cellular apoptosis, proliferation, migration, and survival. Broadly speaking, over-expression of EGFR, which is located on chromosome 7p12, can increase cellular proliferation and contribute to aggressive tumor behavior^31^. Within the molecular context of chordoma, copy number variations at chromosome 7^32^, *i.e.*, gains and partial polysomies, are commonly observed in chordoma, and EGFR gene copy number variations are likewise observed^8^. Several FDA-approved and clinical kinase inhibitors whose main therapeutic target is EGFR have been tested in preclinical in vitro and in vivo models of chordoma^33^. Gefitinib and erlotinib, two FDA-approved EGFR inhibitors used in the treatment of non-small-cell lung carcinoma (NSCLC), individually inhibited U-CH1, U-CH7, UM-Ch-SCor1, and MUG-Chor1 cellular proliferation in dose-dependent manners^20,34^. Afatinib, another FDA-approved EGFR inhibitor used against NSCLC, had anti-proliferative activity against all chordoma cell lines tested (IC_50_ < 0.7 µM) except for JHC7^20^. Afatinib was also found to promote degradation of EGFR and brachyury, both of which are crucial to chordoma cell growth^20^. In vivo studies for erlotinib and afatinib have been reported. Erlotinib treatment significantly lowered tumor volume relative to controls in a PDX mouse model and reduced p-EGFR^9^. Afatinib treatment resulted in tumor growth inhibition in three PDXs and one CDX with no signs of toxicity in any of the mouse models^9^. In addition to this preclinical data, some clinical evidence exists to support EGFR and its therapeutic agents as potential chordoma treatments. A retrospective study found the median PFS was ~ 15.0 months for five patients treated with erlotinib^35^. For afatinib, there is currently an open clinical trial in the Netherlands (NCT03083678) to evaluate its efficacy against locally advanced and metastatic chordoma.

Cyclin-dependent kinase 4 (CDK4) and cyclin-dependent kinase 6 (CDK6) are serine-threonine kinases^36^. CDK4/6 are known oncoproteins, as upregulation of these kinases or inactivation of CDKN2A lead to cell cycle deregulation, increased cell proliferation, and tumorigenesis^37^. CDK4/6 functions downstream of a number of oncogenic pathways, implying that CDK4/6 inhibition may be effective when combined with inhibitors of the upstream pathways^38,39^. Combination treatment of an EGFR sensitive non-small cell lung cancer PDX model with a CDK4/6 inhibitor and an EGFR inhibitor showed combinatorial benefit, providing precedent for a similar application in chordoma tumors which are also sensitive to EGFR inhibition^40^. In another study, palbociclib sensitized lung cancer cells to treatment with EGFR inhibitors^41^. Importantly, in chordoma the CDK4/6 regulatory gene CDKN2A (also known as p16) is frequently lost. CDK4/6 are thus found in an over-activated state in chordoma^42^. From a gene expression perspective, CDK4 and CDK6 mRNAs have been detected in all eight chordoma cell lines that were interrogated: U-CH1, U-CH2, U-CH3, U-CH6, U-CH7, U-CH10, U-CH11, and U-CH12^43^. Likewise, CDK4 and CDK6 protein were observed in the same eight chordoma cell line as above^42^. An immunohistochemistry study of 25 patient samples showed CDK4 was overexpressed in 20% of cases^44^. Tissue microarray analysis of 85 samples from 72 chordoma patients found that ~ 98% of tissue samples expressed CDK4^42^. Additionally, the mean expression level of CDK4 was significantly higher for non-survivors than survivors at the time of publication^42^. There are three FDA-approved kinase inhibitors whose main therapeutic targets are CDK4/6. Palbociclib, one of these dual CDK4/6 inhibitors, has been tested extensively in preclinical in vitro and in vivo models of chordoma^43^. Palbociclib treatment resulted in decreased cell growth in a dose-responsive manner for all eight chordoma cell lines tested^43^. In PDX and CDX models, treatment with palbociclib resulted in significant inhibition of tumor growth in 5/6 individual models (Table S1). There is currently a clinical trial enrolling in Germany (NCT03110744) to evaluate the efficacy of palbociclib against locally advanced and metastatic chordoma.

mTOR is a serine threonine kinase which is a member of two protein complexes, mTOR complex 1 (mTORC1) and complex 2 (mTORC2). By virtue of these interactions it plays key roles in a variety of cellular processes including metabolism and proliferation. It lies in the PI3K–AKT–mTOR signaling pathway, and components of this pathway are implicated in a wide range of cancers. Chordoma is no exception. Analysis of 50 chordomas showed that a high percentage of tumors were positive for p-AKT, piTSC2, p-mTOR, total mTOR, p-P70S6K, p-RPS6, p-4EBPI and eIF-4E^45^. These are all linked to the complicated mTOR signaling pathway. The authors suggested that a majority of chordomas may respond to mTOR inhibitors or mTOR inhibitors in combination with other drugs. A loss of PTEN (a tumor suppressor which negatively regulates this PI3K-AKT-MTOR pathway) was also observed in 16% of these chordoma cases. A study of chordoma tumors from 111 patients demonstrated that three key proteins in the mTOR pathway (p4EBP1, pS6-Ser240/244, pS6-Ser235/236) were activated in 46% of the tumors^46^. In a different study of just skull base chordoma, expression of PI3 and AKT pathway genes was significantly upregulated in brachyury high expression tumors. Importantly, the transcription factor brachyury (gene name *TBXT*), a key driver of chordoma, seems linked to this pathway^47^. The U-CH2 cell line is inhibited by the dual PI3k/mTOR inhibitor BEZ-235, but not the mTORC1-specific inhibitor rapamycin^47^. Given our observations that U-CH2 is inhibited by AZD2014, a highly specific inhibitor of mTOR which inhibits both mTORC1 and mTORC2 activity, we hypothesize that mTORC2 has a critical role in promoting chordoma proliferation or survival. An analysis of sacral chordomas demonstrated that mTOR expression levels were significantly higher than in adjacent normal tissue^48^. The PI3K-mTOR pathway is also upregulated in the UCH-1 chordoma cell line. A PI3K/mTOR inhibitor inhibited both AKT and MTOR activation in this cell line. The compound inhibited proliferation and induced apoptosis^49^. Finally, the MTOR inhibitor MLN0128 (sapanisertib) decreased activity of the PI3K-AKT-MTOR pathway in vivo in a clival chordoma PDX model^50^. Taken together, clearly this pathway is consistently dysregulated in chordomas, and a promising avenue for targeted therapy. Additionally, it has been observed clinically that in one patient, rapamycin, an FDA-approved mTOR inhibitor, slowed the progression of a recurrent chordoma tumor^51^. The utility of these various kinase inhibitors in preclinical and clinical chordoma settings implies that multiple oncogenic biological pathways may drive chordoma.

Based on these observations, we hypothesized that combinations of clinical EGFR, CDK, and mTOR inhibitors may act synergistically by dampening oncogenic signaling in multiple pathways. Using a combination of computational and in vitro approaches we have identified AZD2014 (targets mTORC1 and mTORC2), RDEA119 (targets MEK1/2), and AZD4054 (targets endothelin A receptor) as compounds which inhibit chordoma cell lines to differing extents. We also assessed several kinase drug combinations, of these, afatinib (EGFR) and palbociclib (CDK4/6) as well as afatinib and AZD2014 showed substantial synergy against U-CH1 cells in vitro. Since each of these pairs of compounds likely have kinome inhibition profiles that are distinct from one another^19^, co-dosing will allow us to target two key chordoma vulnerabilities simultaneously. Another advantage of working with these compounds is that pharmacokinetics data for in vivo studies is available^52-55^. There is a growing body of data on the effects of small molecules on the growth of chordoma cell lines^20,21^. The results of our studies indicate that machine learning approaches utilizing these data^20,21^ can help identify compounds suitable for testing in chordoma cell line models, and that we have identified combinations of inhibitors that act on different pathways important for chordoma cell growth which can behave synergistically. This work paves the way for future in vivo evaluation of CDK / mTOR inhibitor combinations in animal models of chordoma and then in the clinic. A treatment for chordoma would fill a dire unmet clinical need, and repurposing of available medicines is likely the quickest approach to approved treatments.

## Methods

### Compounds

Palbociclib was obtained from Apexbio (Houston TX). Afatinib, AZD0530, RDEA11, AZD4054 and AZD2014 were obtained from Selleckchem (Houston, TX). Compounds were dissolved in DMSO, before dilution in cell culture media for cell-based assays.

### In vitro testing

All chordoma cell lines were cultured in Iscove's Modified Dulbecco's Medium (IMDM): Roswell Park Memorial Institute (RPMI) in a 4:1 ratio (Gibco, Life Technologies, NY, USA) and 10% fetal bovine serum (Seradigm, VWR, USA) at 37 °C, 5% CO_2_ in 0.1% gelatin coated flasks. There were no media antibiotics used. Cell lines were then seeded with approximately 800 cells/well in 384 well gelatin coated plates and allowed to adhere overnight before the addition of drugs. Drugs were added in quadruplicate wells using a Tecan EVO150 with 96 MCA head (Tecan Group Ltd, Switzerland). Each plate included controls for drug solvation effects from DMSO. Plates were incubated with drugs for 48hrs prior to the addition of resazurin substrate (Alamar Blue, Biosource International, Camarillo California). Plates were then incubated for a further 18hrs before reading using an Infinite F200 microplate reader with a Connect Stacker (Tecan Group Ltd). iControl software (Version 1.11) was then used to measure the fluorescence intensity of resarufin at EX535nm and EM595nm. The resulting relative fluorescence units are proportional to cellular redox activity, which is a common proxy for the quantity of living cells^56^.

After adjusting for the effects of DMSO in vehicle-only controls, raw fluorescence data was fitted to a four-parameter log-logistic dose response model. This model was constrained to fit a common upper asymptote for each individual experiment, and a positive lower asymptote. Each experiment was repeated for a total of 2–7 biological replicates, and estimated IC_50_′s and maximal effects were combined using geometric and arithmetic means, respectively.

### Combination testing

For synergy experiments, drugs were tested in combination using a fixed-ratio “ray” design^57^. Drugs were diluted to 10 × previously estimated IC_50_ and combined in specific ratios (80%:20%, 50%:50% and 20%:80%). These combined drugs were serially diluted to maintain a constant ratio. Dose–response curves were fit for each mixture of drugs. Data from multiple biological replicates was combined in a mixed-effects model, using the function metadrc() in the R package medrc.^58^ Absolute IC_50_ estimates from these models were then used to calculate the combination index CI = a/A + b/B, where A and B are doses of individual drugs that produce a specified effect, and (a, b) is the pair of doses in a combination that produces the same effect^59^. Confidence intervals of CI were estimated using confidence limits of each IC_50_ estimate in the same equation. CI < 1 indicates synergy, CI = 1 no effect, and CI > 1 antagonism.

### Software for machine learning

Chordoma datasets were named as Broad^21^ and EGFR^20^. These datasets consist of molecules screened against multiple cell lines and the original authors provided potency or activity classifications which were used as a binary score. These datasets underwent curation to remove problematic molecules before model building as described elsewhere^10,12,22-29^. We utilized Assay Central which has been previously described in detail^10,12,22-29^ to prepare and merge datasets collated in Molecular Notebook^60^, as well as generate Bayesian models using ECFP6 descriptors^61,62^. Briefly, the Assay Central project includes automated workflows for curating well-defined structure–activity datasets that employ a set of rules for the detection of problematic data (i.e. abnormal valences, multiple components) that can be corrected by multiple means. Data that is compatible with machine learning is then used to generate a Bayesian model for prospective bioactivity predictions. Each Bayesian model in Assay Central includes the following metrics generated from fivefold cross validation: Recall, Precision, Specificity, F1-Score, Receiver Operating Characteristic (ROC) curve, Cohen’s Kappa (CK)^63,64^, and the Matthews Correlation Coefficient (MCC)^65^. Assay Central prediction workflows assign a probability-like score^61,62^, with values above 0.5 considered an active prediction, and an applicability score which assesses the portion of fragments overlapping with the training set molecules to the input compounds. Predictions were applied to the specific compounds of interest from NCATS (https://web.archive.org/web/20170716163452/https:/ncats.nih.gov/ntu/assets/2017#Adult_Indications ) that were not in the training sets (AZD0530 was in the Broad dataset and was therefore not scored by the models) prior to in vitro testing as we have done previously^25^.

## Supplementary information

Supplementary Information.
