# Supplementary material for: Synergistic drug combinations and machine learning for drug repurposing in chordoma
Source: Sci Rep. 2020 Jul 31;10:12982. doi: 10.1038/s41598-020-70026-w (PMC7395084; doi:10.1038/s41598-020-70026-w)

**Synergistic Drug Combinations and Machine Learning for** **Drug Repurposing in Chordoma**

Edward Anderson^1^, Tammy Havener^1^, Kimberley Zorn^2^, Daniel Foil^2^, Thomas Lane^2^, Stephen J. Capuzzi^1^, Dave Morris^1^, Anthony J. Hickey^1, 3^, David H. Drewry^4^ and Sean Ekins^1,2^

^1^UNC Catalyst for Rare Diseases, Eshelman School of Pharmacy, University of North Carolina at Chapel Hill, North Carolina, United States of America

^2^Collaborations Pharmaceuticals, Inc., 840 Main Campus Drive, Lab 3510, Raleigh, North Carolina, United States of America.

^3^RTI International, Research Triangle Park, North Carolina, United States of America

^4^Structural Genomics Consortium, UNC Eshelman School of Pharmacy, University of North Carolina at Chapel Hill, Chapel Hill, NC 27599, USA

* To whom correspondence should be addressed. E-mail: [sean@collaborationspharma.com](mailto:sean@collaborationspharma.com)

Phone: 215-687-1320

**Running Head**: Repurposing for chordoma

**Table S1. Kinase inhibitor testing in mouse models.** Afatinib and palbociclib were individually tested in six separate PDX/CDX chordoma mouse models (data from The Chordoma Foundation ^33^, afatanib data recently published ^9^ palbociclib unpublished). Afatinib demonstrated significant (p-value < 0.05) tumor growth inhibition relative to control vehicle for all six models. Palbociclib demonstrated significant (p-value < 0.05) tumor growth inhibition relative to control vehicle for five out of six models. Afatinib was dosed at 20 mg/kg by mouth (po); either once a day (qd) to the end of the study or qd x 28 days. Palbociclib was dosed at 75 mg/kg po once a day to the end of the study. Results of patient-derived and cell line-derived Xenografts for afatinib and palbociclib. Afatinib, an EGFR inhibitor, and palbociclib, a CDK4/6 inhibitor, were tested in a total of 6 xenograft mouse models (2 CDX and 4 PDX). Percentage of tumor growth inhibition (%TGI).

| **Drug** | **Source** | **Xenograft** | **% TGI** | **P-value** |
| --- | --- | --- | --- | --- |
| Palbociclib | CF322 | PDX | 71 | 0.0071 |
| Afatinib | CF322 | PDX | 92 | 0.0007 |
| Palbociclib | CF359 | PDX | 54 | 0.0662 |
| Afatinib | CF359 | PDX | 33 | 0.3843 |
| Palbociclib | CF365 | PDX | 87 | 0.0001 |
| Afatinib | CF365 | PDX | 100 | 0.0001 |
| Palbociclib | CH22 | CDX | 61 | 0.0029 |
| Afatinib | CH22 | CDX | 56 | 0.0066 |
| Palbociclib | SF8894 | PDX | 59 | 0.0001 |
| Afatinib | SF8894 | PDX | 87 | 0.0001 |
| Palbociclib | U-CH1 | CDX | 100 | 0.0106 |
| Afatinib | U-CH1 | CDX | 100 | 0.0007 |
|  |  |  |  |  |

**Figure S1. ROC plots for chordoma models.** A. Broad, B. EGFR, C Broad+EGFR. Datasets were named as Broad ^21^ and EGFR ^20^, and underwent curation to remove problematic molecules before model building. Data represent 5-fold cross validation.

A.
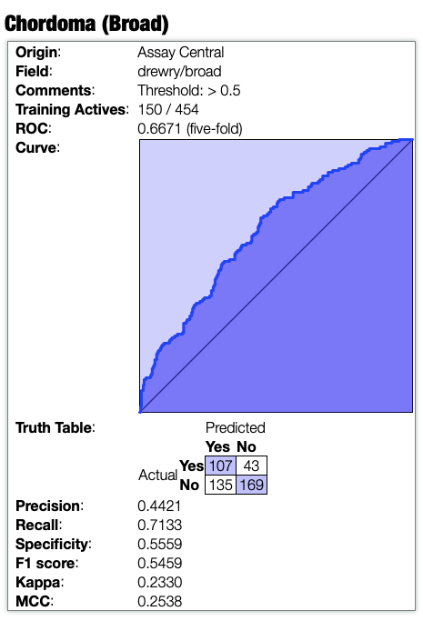
B.
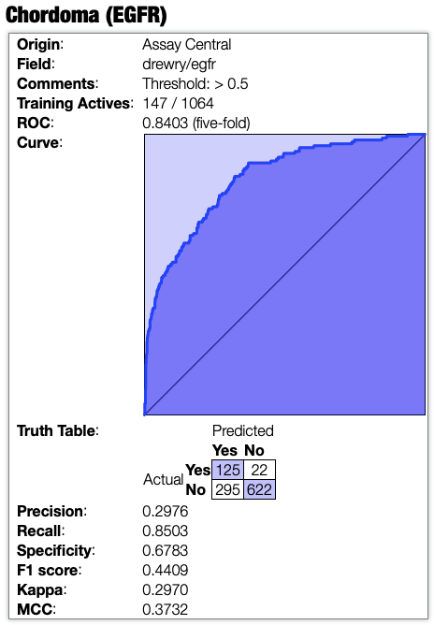
C.
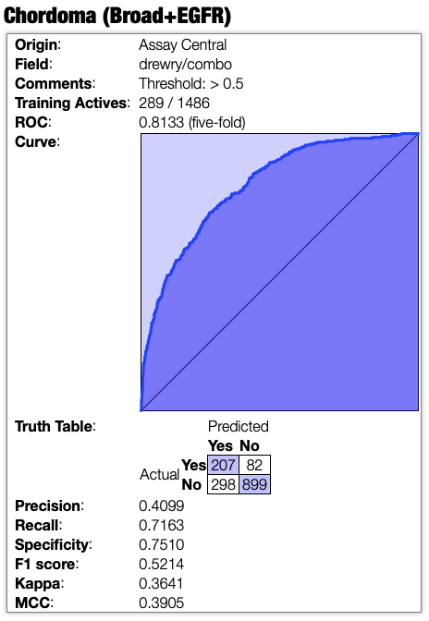

Supplement: Supplementary file 1 — Supplementary Information. [file 41598_2020_70026_MOESM1_ESM.docx]
